# Plasma Rich in Growth Factors Induces Cell Proliferation, Migration, Differentiation, and Cell Survival of Adipose-Derived Stem Cells

**DOI:** 10.1155/2017/5946527

**Published:** 2017-11-15

**Authors:** Maravillas Mellado-López, Richard J. Griffeth, Jose Meseguer-Ripolles, Ramón Cugat, Montserrat García, Victoria Moreno-Manzano

**Affiliations:** ^1^Tissue and Neuronal Regeneration Laboratory, Centro de Investigación Príncipe Felipe, Valencia, Spain; ^2^Unidad de Artroscopia y Unidad de Traumatología del Hospital Quiron, Barcelona, Spain; ^3^Fundación García Cugat, Barcelona, Spain; ^4^FactorStem Ltd., Valencia, Spain

## Abstract

Adipose-derived stem cells (ASCs) are a promising therapeutic alternative for tissue repair in various clinical applications. However, restrictive cell survival, differential tissue integration, and undirected cell differentiation after transplantation in a hostile microenvironment are complications that require refinement. Plasma rich in growth factors (PRGF) from platelet-rich plasma favors human and canine ASC survival, proliferation, and delaying human ASC senescence and autophagocytosis in comparison with serum-containing cultures. In addition, canine and human-derived ASCs efficiently differentiate into osteocytes, adipocytes, or chondrocytes in the presence of PRGF. PRGF treatment induces phosphorylation of AKT preventing ASC death induced by lethal concentrations of hydrogen peroxide. Indeed, AKT inhibition abolished the PRGF apoptosis prevention in ASC exposed to 100 *μ*M of hydrogen peroxide. Here, we show that canine ASCs respond to PRGF stimulus similarly to the human cells regarding cell survival and differentiation postulating the use of dogs as a suitable translational model. Overall, PRGF would be employed as a serum substitute for mesenchymal stem cell amplification to improve cell differentiation and as a preconditioning agent to prevent oxidative cell death.

## 1. Introduction

Adipose-derived stem cell (ASC) transplantation has already demonstrated effectiveness and continues to be an important avenue of research and development due to their extraordinary therapeutic aptitude. ASC transplantation has shown remarkable restorative ability reflected in the significant recovery of function in patients with a range of diseases such as Parkinson's, Alzheimer's, spinal cord injury, heart disease, and rheumatoid arthritis [1]. Nevertheless, new approaches for ASC isolation and amplification have been developed [2–4], to generate sufficient amount of stem cells to optimize clinical applications [5]. However, the paucity of information regarding ASC survival after transplantation lends itself to further investigation of ASC quantity and quality before transplantation. For instance, ASC activation with defined stimuli prior transplantation may enhance ASC repair capabilities and improve success rates for regenerative treatments. Recent studies have focused on increasing the yield, efficiency, and therapeutic capability of ASC by treating them with growth factors like PDGF or bFGF [6–8] or as a xeno-free alternative for mesenchymal stem cell expansion [5, 9]. A major source of endogenous growth factors is the plasma rich in growth factors (PRGF) from platelet-rich plasma [10]. PRGF has been extensively used in many species to reduce healing time and to improve tissue regeneration [7, 11]. PRGF beneficial effects are modulated by the degranulation of alpha granules in platelets [12, 13] which contain several important growth factors that stimulate cell growth, proliferation, and differentiation [14, 15]. PRGF stimulates undifferentiated stem cells to proliferate and differentiate and has been used for tissue regeneration [8, 16–18] purposes. Undifferentiated stem cells migrate to the concentration of platelet-releasing growth factors triggering proliferation of the cells at the site [19]. Moreover, platelet-derived growth factors enhance sternness of ASC [20] being proposed as a gold standard fetal bovine serum replacing method for human cell propagation for clinical applications [5, 21, 22]. Additionally, PRGF has also shown synergistic properties on mesenchymal stem-induced differentiation which accelerate bone [23] or cartilage repair [24].

Minimizing aging of ASC cultures represents a significant challenge for tissue engineering especially for autologous cell-based approaches in geriatric patients [25]. New approaches have been considered to avoid ASC senescence for prolonged ex vivo expansion or induced differentiation avoiding, for instance, hypertrophic phenotypes on chondrocytes-induced differentiation process [26], by forcing the expression of hTERT, and telomerase activity induction, to extend the lifespan of the mesenchymal cells on culture. Previous reported data shows that hTERT-transduced mesenchymal stem cells have prolonged replicative capacity in vitro and keep the adipo-, chondro-, and osteogenic differentiation potential in vitro and osteogenic potential in vivo [27, 28]. Importantly, although there was no evidence for tumor formation or cell transformation, nongenetic manipulations will be more suitable for further potential clinical applications.

The serine/threonine kinase AKT represents an initial signaling node within all cells of higher eukaryotes contributing significantly to the regulation of survival, growth, proliferation, angiogenesis, and metabolism of many cell types [29]. AKT activation by PRGF enhances survival and regenerative function in ASC [30]. Furthermore, conditioning of ASC by inducing AKT activity increases cell survival and proangiogenic capacities [31]. The induction of cell survival signals, such as AKT, would also confer a resistance to hostile environments such as those associated with an inflammatory response, which induce cell death by oxidative stress [32].

## 2. Material and Methods

### 2.1. ASC Isolation and Culture

Adipose tissue was collected from dogs and humans, both of which were suffering from osteoarthritis, and was performed in an operating room by veterinarians and physicians, respectively. A 10 g biopsy of subcutaneous fat from dog patients [8] or from the suprapatellar fat pad from human patients [33] was collected, *n* = 4 (dogs) and *n* = 4 (humans). All procedures were performed under sterile conditions, and the adipose tissue was placed into sterile conical tubes containing sterile saline. The experimental procedures for dogs did not require evaluation by the animal Ethics Committee because the procedure only included a cession of part of the amplified ASCs needed for cell transplantation, and for this purpose, the canine owners voluntarily signed an informed consent for the use of surplus adipose tissue utilized for the derivation of ASCs and further research purposes. The human samples were anonymized, and this experimental procedure has been evaluated and accepted by the Regional Ethics Committee for Clinical Research with Medicines and Health Products following the Code of Practice 2014/01. As exclusion criteria, no samples were collected from patients with a history of cancer or infectious diseases at the time of the surgery (viral or bacterial). All human patients voluntarily signed an informed consent document for the use of surplus adipose tissue and donation of peripheral blood (20 ml) collected sodium citrate containing tubes for PRGF isolation prepared following the standardized method described in Anitua et al. [14], pooled to minimize differences between individuals and stored at −20°C.

Adipose tissue was transferred from the surgery room in an enclosed package at 4°C in sterile solution and arrived at the laboratory within 24 h after extraction. Each sample was washed multiple times in PBS plus antibiotics to clean the tissue and remove residual blood. Adipose tissue was then placed into sterile Petri dishes (10 g adipose tissue per 100 mm Petri dish), in a solution containing PBS, 100 units/ml penicillin and 100 *μ*g/ml streptomycin (Gibco 15140), collagenase type I-A (0.07%, Sigma-Aldrich C9891 CA, USA), and dispase I (0.2 mM, Sigma-Aldrich). The adipose tissue was manually cut into small pieces using sterile surgical scissors in a laminar flow hood and transferred to a cell flask for overnight digestion in a shaker at 37°C, 20% O_2_, and 5% CO_2_. On the following day, the digested adipose tissue was collected and washed multiple times with PBS plus antibiotics by serial centrifugation. The cell pellet was then resuspended in growth medium (DMEM containing 2 mM L-glutamine, 30% L-glucose, 100 units/ml penicillin, and 100 *μ*g/ml streptomycin), plus 10% fetal bovine serum (FBS) for canine samples or 10% human serum (HS) for human samples. On the following day, the medium was removed and replaced with fresh medium and attached cells were allowed to grow until nearly confluent and then subjected to cell proliferation, survival studies, and cell differentiation assays.

### 2.2. Cell Proliferation Assay

At passages 3-4, canine and human ASCs, 10^4^ cells were seeded in 24-well plates in the presence of 10% of FBS or HS, respectively, for 24 hours. Then, the medium was replaced for 0, 1, 2.5, 5, or 10% of FBS or HS or PRGF-containing medium and cells were maintained on culture for 24 hours incubation. For cell quantification, each well was trypsinized and counted in a Neubauer® chamber. Three independent experiments were performed in triplicates. Data was expressed as mean ± SD.

### 2.3. Cell Migration and Invasion Analysis by In Vitro Scratch Assay

Cell migration for wound healing of the induced in vitro scratch was performed in the IncuCyte® S3 live-cell analysis [34, 35]. 15 × 10^5^ ASCs were seeded in 96-well plates for a monolayer cell culture in the presence of 10% of HS. 24 hours after seeding, the scratch was performed at the time in all wells by using the WoundMaker tool. Then, the medium was replaced by 0% HS or PRGF, 2.5%, or 10% of HS or PRGF in triplicates. Cell growth and migration were monitored under phase-contrast microscopy connected to a time-lapse recording system at the scratched area, every hour during 16 h. A picture from four different fields at each experimental condition was recorded for time-lapse reconstruction and to quantify the cell density of repopulated scratched area. Image analysis was automatically performed by the associated software by following the reported mathematical analysis in [35]. The results are shown as the mean ± SD of the % of relative wound density at every experimental condition.

### 2.4. Senescence-Associated Beta-Galactosidase (SA*β*Gal) Activity

Cells were seeded at 2000 cells/cm^2^ in six-well plates in the presence of 10% of HS. 24 hours after seeding, the medium was replaced for 2.5% PRGF, 2.5% HS, 10% HS, or 0% of PRGF or HS. 48 hours after incubation, cells were fixed in 4% paraformaldehyde for 10 minutes and assayed for SA*β*Gal activity as described by Debacq-Chainiaux et al. [36]. SA*β*Gal-positive cells were counted with a minimum of 200 cells overall for each condition. Three independent experiments were performed. Data was expressed as the mean ± SD of the percentage ratio of *β*Gal-positive cells within the total assayed cell culture.

### 2.5. Transmission Electron Microscopy and Autophagosome Quantification

Cells were seeded at 2000 cells/cm^2^ in Lab-Tek chamber slides (Nalge Nunc International, Naperville, IL) in the presence of 10% of HS. 24 hours after seeding, the medium was replaced for 2.5% PRGF, 2.5% HS, 10% HS, or 0% of PRGF or HS. 48 hours after incubation, cells were fixed in 3% glutaraldehyde for 1 hour at 37°C. Cells were post fixed in 2% OsO_4_ for 1 hour at room temperature and stained in 1% uranyl acetate in the dark for 2 h at 4°C. Finally, cells were rinsed in distilled water, dehydrated in ethanol, and infiltrated overnight in Durcupan resin (Fluka, Sigma-Aldrich, St. Louis, USA). Following polymerization, embedded cultures were detached from the chamber slide and glued to araldite blocks. Serial semithin sections (1.5 *μ*m) were cut with an Ultra cut UC-6 (Leica, Heidelberg, Germany) and mounted onto slides and stained with 1% toluidine blue. Selected semithin sections were glued with Super Glue-3, Loctite (Henkel, Düsseldorf, Germany) to araldite blocks and detached from the glass slide by repeated freezing (in liquid nitrogen) and thawing. Ultrathin sections (0.06–0.08 *μ*m) were prepared with the Ultra cut and stained with lead citrate. Finally, photomicrographs were obtained under a transmission electron microscope FEI Tecnai G2 Spirit (FEI Europe, Eindhoven, Netherlands) using a digital camera Morada (Olympus Soft Image Solutions GmbH, Münster, Germany). Autophagosomes were morphologically identified (^∗^) and quantified from at least 10 different pictures, at equal magnification, for each experimental condition and normalized to the total cell area by using the ImageJ software in pixels (px^2^). Three independent experiments were performed, and data was expressed as mean ± SD of autophagosomes/total cell area (px^2^).

### 2.6. ASC-Directed Differentiation

Confluent canine and human ASCs at passage 4 were subjected to directed differentiation towards adipocytes, osteocytes, or chondrocytes [37]. All three differentiation processes were performed in parallel with 2.5% thawed PRGF plus heparin (40 U) or heparin alone. All directed differentiation media were obtained from Lonza Group Ltd.

#### 2.6.1. Adipogenesis

ASCs were seeded at a cell density of 10,000 cells/cm^2^, and when ASCs were >90% confluent, the growth medium was changed to differentiation medium containing insulin, dexamethasone, IBMX (3-isobutyl-methyl-xantine), and indomethacin (adipose-derived stem cell Basal Medium; Lonza Group Ltd.). The cells were then incubated for 10–12 days. Adipogenic differentiation was evaluated by Oil Red O staining of the lipid vacuoles in formalin-fixed cultures.

#### 2.6.2. Osteogenesis

ASCs were seeded at a cell density of 10,000 cells/cm^2^ in collagen I-coated plates (Sigma-Aldrich; 10 mM) in medium containing 0.1 *μ*M dexamethasone, 50 *μ*M Asc2P, and 10 mM *μ*-glycerophosphate (osteogenic basal medium; Lonza Group Ltd.) with 10% human serum. ASC cultures were maintained in this medium for 4 weeks (with medium changes every 3 days). For detection of extracellular calcium deposits, Alizarin Red staining was used in formalin-fixed cultures. Immunodetection, by immunofluorescence, was performed to detect actin filaments by phalloidin and osteocytes by Connexin 43.

#### 2.6.3. Chondrogenesis

Differentiation of chondrocytes was performed using a micromass. Starting with a high concentration of ASC in a minimal volume (1 × 10^5^ cells/100 *μ*l) in the presence of TGF-*β* 1 and 3 (10 ng/ml), Asc 2P (50 *μ*M), and insulin (6.25 *μ*g/ml) (Chondro BulletKit; Lonza Group Ltd.), these ASCs were cultured for four weeks in this medium, with medium changes every 3 days. Alcian blue was used to detect the presence of enrichment of sulfated proteoglycans in the extracellular matrix. Before staining, the micromass cultures were fixed in formalin, embedded in paraffin, and cut into 5 *μ*m sections.

### 2.7. Immunocytochemistry

ASC monolayer was fixed with 4% PFA at room temperature for 15 minutes, permeabilized with 0.1% Triton X-100, and subsequently blocked with 10% FBS. Micromass sections from chondrocyte-induced differentiation were previously dewaxed. Incubation with one of the following primary antibodies was performed overnight (1 : 200) at 4°C: Connexin 43 (Abcam; UK); Sox9 (Chemicon, USA). After removing primary antibodies and washing thoroughly, one of the following secondary antibodies (1 : 400) was added and incubated for 1 h at room temperature; Oregon green 488 goat anti-mouse IgG or Alexa Fluor 647 mouse anti-rabbit (Thermo Fisher Scientific, USA). Phalloidin conjugated with FITC (1 : 1000; Invitrogen, USA) was incubated for 45 minutes before visualization. All cells were counterstained by incubation with 4,6-diamidino-2-phenylindole dihydrochloride (DAPI) from Molecular Probes (Invitrogen, USA) for 3 min at room temperature followed by washing steps. Samples were mounted using FluorSave Reagent (Calbiochem, USA). Confocal Microscopy (Leica, Germany) was employed to visualize the signals; at least six different fields per condition and assay were analysed.

### 2.8. ASC Treatments and Survival Studies in the Presence of Hydrogen Peroxide

The H_2_O_2_ (Sigma-Aldrich, USA) and AKT inhibitors (Calbiochem, USA; AKT inhibitor VIII 124018; 10 *μ*M) were freshly prepared from 1 M and 10 mM stock solutions, respectively. PRGF and H_2_O_2_ combined treatments were incubated at the same time. AKT inhibitor was preincubated for 30 minutes prior to secondary treatments.

The cell viability was determined by the CellTiter 96® AQueous Non-Radioactive Cell Proliferation Assay (Promega Co., Madison, WI, USA) following the manufacturer's instructions. Briefly, 10^5^ ASCs at passages 2–5 were seeded onto 96-well plates and allowed to grow for 24 h in growth medium. After removing the growth medium, the cells were treated with PRGF (0 or 2.5%, plus 40 U of heparin), 2.5% of HS, H_2_O_2_ (100 *μ*M), and/or AKT inhibitor (Calbiochem, USA; AKT Inhibitor VIII 124018; 10 *μ*M) for 24 h in the absence of serum. Every condition was assayed in quadruplicate in three different experiments. The viability of cells at each assayed condition was expressed as the percentage ratio of the mean ± SD.

### 2.9. Annexin V Detection by FACS Analysis

ASCs were trypsinized, and 10^5^ cells per condition were diluted into 100 *μ*l of PBS and incubated with 1 : 50 dilution of annexin V-FITC-conjugated antibody (Invitrogen, USA) in the dark for 45 min at room temperature and then washed three times with PBS and resuspended in 0.3 ml of cold PBS for flow cytometry analysis (FC500, Beckman Cultek, USA). The mean ± SD of the annexin V-positive population (in percentage) of all tested samples were represented.

### 2.10. Western Blotting Analysis

ASCs were collected, and proteins were extracted by using Lysis Buffer (50 mM Tris-HCl, pH 7.5, 150 mM NaCl, 0.02% NaN_3_, 0.1 SDS, 1% NP40, 1 mM EDTA, 2 *μ*g/ml leupeptin, 2 *μ*g/ml aprotinine, 1 mM PMSF, and 1x Protease Inhibitor Cocktail (Roche Diagnostics, USA)). Equal amounts of protein extracts (20 *μ*g) were loaded onto a 10% SDS-polyacrylamide gel and resolved by standard SDS-PAGE. Proteins were electrophoretic transferred onto PVDF membranes. Membranes were blocked with 5% skim milk in PBST for 60 min and incubated overnight with one of the following specific primary antibodies (1 : 1000): PARP (Abcam, UK); P-AKT, AKT, or Cyclin D (Cell Signaling, USA). *β*-Actin (1 : 5000) (Sigma-Aldrich, USA) was used as a loading control. Subsequently, membranes were incubated with rabbit anti-mouse or rabbit anti-goat horseradish peroxidase-conjugated secondary antibody (1 : 5000) (Sigma-Aldrich, USA). Blots were visualized by the ECL detection system (Amersham, UK).

### 2.11. Statistical Analysis

Statistical comparisons by pairs were assessed by Student's *t*-test. All *P* values were derived from a two-tailed statistical test using the SPSS 11.5 software. A value of *P* < 0.05 was considered statistically significant.

## 3. Results

### 3.1. PRGF Induces Proliferation and Migration of ASCs

**Figure 1:**
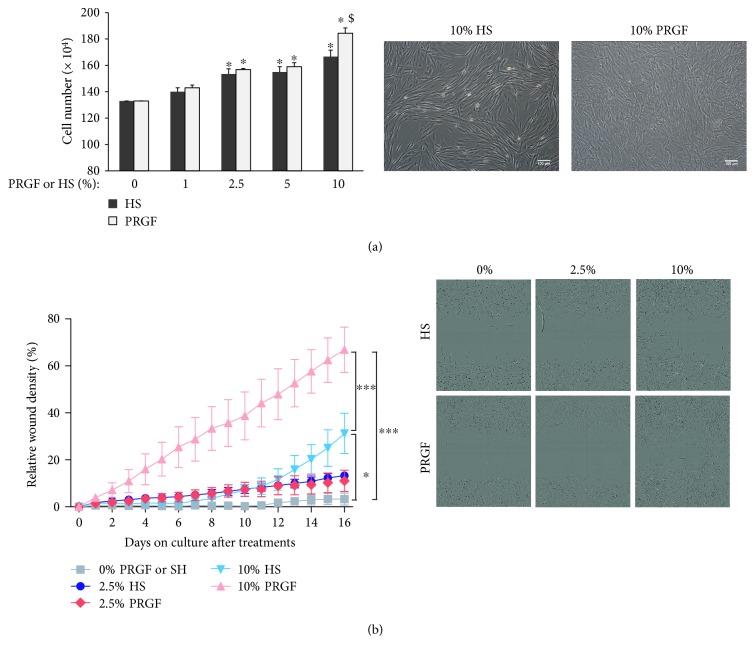
PRGF induces proliferation and migration of human ASCs. (a) Left: Human ASCs were cultured with HS or PRGF at growing concentration (1, 2.5, 5, or 10%) or in the absent of growth factors (0%) for 24 hours and subjected to cell viability test analysis. 10% PRGF induced a significant difference on the cell numbers in comparison with 10% of HS; ^∗^*P* > 0.05 versus 0% and ^$^*P* > 0.05 versus 10% HS; Right: representative phase-contrast images of ASCs 24 hours after incubation with 10% HS or 10% PRGF; scale bar: 100 *μ*m. (b) Cell migration and invasion assay was performed in 96-well plates by the IncuCyte S3 live-cell analysis. Left: cell density quantification at the wound area showed a faster and significant increase of cell density induced by 10% of PRGF since 2 hours after stimulation. 10% HS significantly induced the cell migration and invasion in comparison with 0% since 10 hours of incubation. ^∗∗∗^*P* > 0.001; ^∗^*P* > 0.05. Right: representative phase-contrast images of human ASCs in the presence of different concentrations of HS or PRGF 16 hours after incubation.

Human ASCs in the presence of growing concentrations of HS or PRGF (1, 2.5, 5, or 10%), for 24 h, exhibited significant increased proliferation in comparison with absent of growth factors (0%; Figure 1(a), left graph; ^∗^*P* > 0.05). 10% of PRGF induced the highest proliferation rates and was significantly different to the HS proliferative activity (Figure 1(a), left graph; ^$^*P* > 0.05). Representative phase-contrast images of human ASCs in the presence of 10% HS or PRGF are shown in Figure 1(a) (right). Similarly, in a cell invasion scratch assay, 10% of PRGF induced the highest cell migration activity, significantly different in comparison with ASC in the presence of HS (Figure 1(b), left graph). Representative photograms from time-lapse analysis, 16 hours after PRGF or HS treatments, evidenced both the increase of cell density and the accelerated wound invasion induced by 10% PRGF (Figure 1(b), right panels). Canine ASCs showed comparable respond to human ASCs. 10% of canine PRGF induced higher proliferation rates in comparison with FBS containing canine ASC cultures (Supplementary Figure 1 available online at https://doi.org/10.1155/2017/5946527).

### 3.2. PRGF Reduces Senescence and Autophagocytosis of In Vitro Expanded ASC

**Figure 2:**
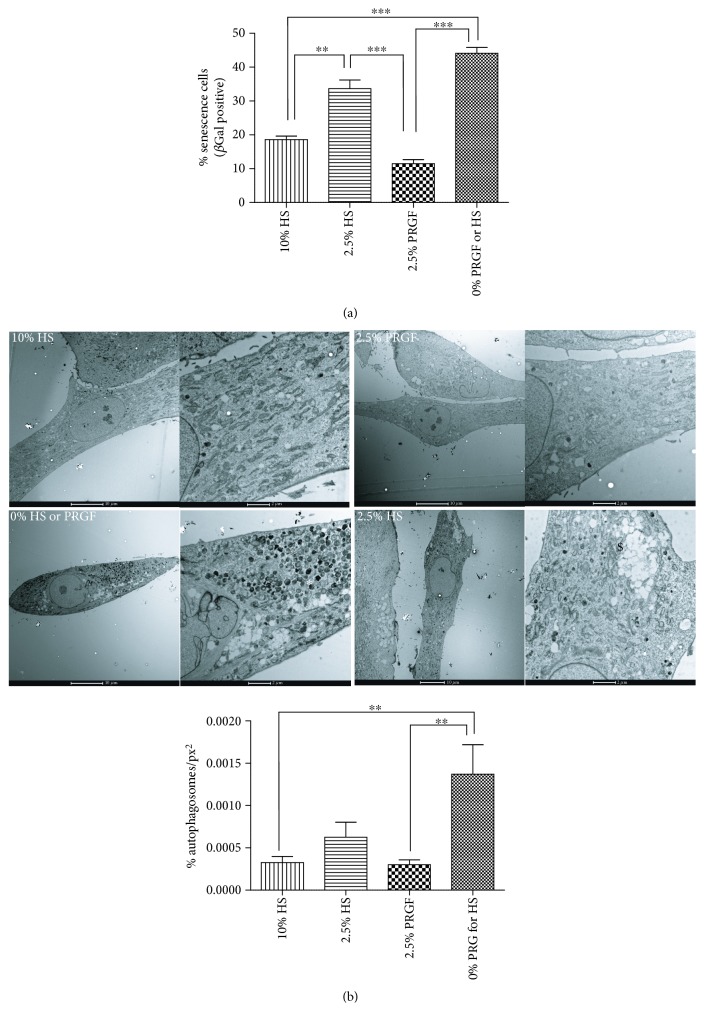
PRGF prevents in vitro ASC aging. (a) Senescence cell quantification was performed by quantification of SA*β*Gal-positive cells in bright field microscope at 20x magnification. Four different fields, containing a minimum of 200 cells, were analysed in three independent experiments. (b) Autophagosome quantification was performed from higher magnification TEM pictures from at least 200 different cells. The total number of autophagosomes was normalized to the total area analyzed with ImageJ software. Three independent experiments were analysed and represented as the mean ± SD of autophagosomes/total cell area (px^2^) ^∗∗^*P* > 0.01; ^∗∗∗^*P* > 0.0001.

Ex vivo amplification of ASCs has shown to be limited to a certain number of passages constituting a limitation for generation of sufficient high cell quantities on clinical usage. Indeed, cellular alterations occurring during in vitro aging have been suggested to be similar to differences observed on ASCs from aged and young donors [38]. There is increasing evidence that cellular senescence is a cause of stem cell aging and malignancy [39]. Herein, we report that 2.5% of PRGF reduces senescence of human ASCs upon starvation, in the absence of growth factors, but not the equivalent concentration of HS, in comparison with the total absent of growth factors (Figure 2(a)). Similarly, the significant accumulation of autophagosomes, a hallmark of cell aging [40], induced by the lack of growth factors, was significantly prevented with 2.5% of PRGF and not with the same concentration of HS (Figure 2(b); graph). Transmission electron microscopy representative images show an extensive accumulation of autophagosome bodies (∗) and vacuoles ($) in the complete absence of growth factors and in less extend, but also significant, in the ASCs in the presence of 2.5% of HS (Figure 2(b); upper panels). A dose response of PRGF on reducing senescence and autophagocytosis was assayed also for 5 and 10% of PRGF, and no significant differences were found in comparison with 2.5% PRGF (data not shown).

### 3.3. PRGF Accelerates ASC-Directed In Vitro Differentiation of Adipocytes, Osteocytes, and Chondrocytes

**Figure 3:**
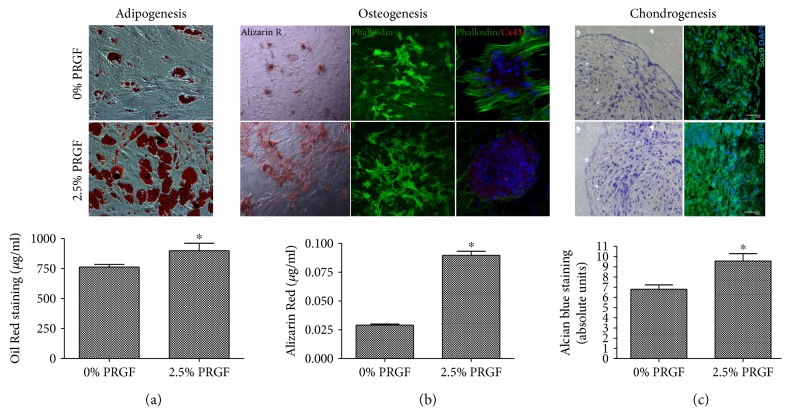
PRGF accelerates adipocyte, osteocyte, and chondrocyte in vitro differentiation of human ASCs. Human ASCs were induced to differentiate toward the three mesodermal lineages in the presence or absence of 2.5% PRGF; (a) adipogenesis: the presence of PRGF induced higher intracellular lipid content by Oil Red O staining; (b) osteogenesis: both calcium deposits visualized by Alizarin Red staining and the phalloidin (green: which indicates actin cytoskeleton growth in osteocytes) and Cx43 expression (red: a known marker of mature osteocytes) were improved in the presence of PRGF; (c) chondrogenesis: Alcian blue staining (marker of the proteoglycan aggrecan deposits) and immunostaining of Sox9 (green: marker of chondrogenesis and chondrocyte differentiation) were higher in the presence of PRGF. Scale bar: 50 *μ*m. ^∗^*P* < 0.05 versus 0% PRGF.

Human ASCs in the presence of PRGF differentiated more rapidly toward adipocytes, osteocytes, and chondrocytes (Figures 3(a), 3(b) and 3(c)). Human ASCs were induced to differentiate into the three mesodermal lineages, in the presence or absence of 2.5% PRGF in the defined differentiated mediums for each lineage. Directed adipogenesis revealed a more rapid accumulation of fat-containing cells in the presence of PRGF, as visualized and quantified by the Oil Red O staining (Figure 3(a)). Osteocyte differentiation in the presence of PRGF was even more robust and accelerated. PRGF treatment leads to increased expression of Cx43, a known marker of mature osteocytes, at four weeks of the differentiation process and a higher number of calcium containing osteocyte cell clusters as demonstrated by Alizarin Red staining quantification (Figure 3(b)). ASCs differentiated toward chondrocytes showed higher Sox9 expression in the presence of PRGF (Figure 3(c), right panels). In addition, cell number and density increased in the presence of PRGF, with an enrichment of collagen in the micromass cultures for chondrocyte induction as visualized and quantified by Alcian blue staining (Figure 3(c), left panels). Canine PRGF also improved the yield on the directed differentiation process into adipocytes, osteocytes, and chondrocytes as shown in the representative images of Supplementary Figure 2.

### 3.4. PRGF Exhibits Improved Tolerance to Hydrogen Peroxide Cytotoxicity in Human ASCs by AKT Induction

**Figure 4:**
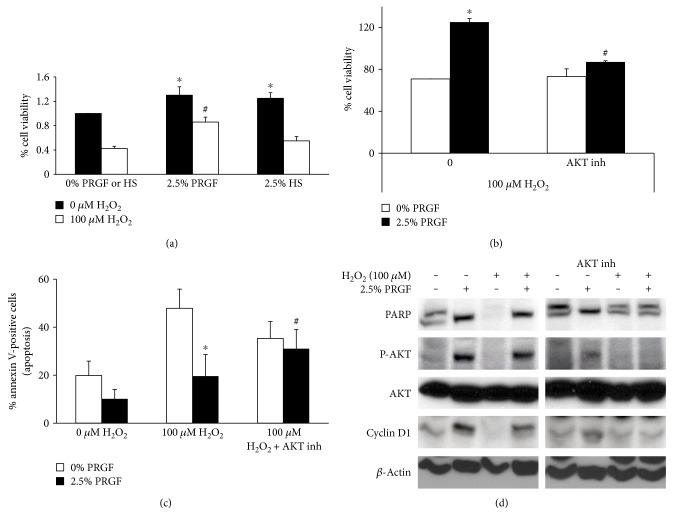
AKT mediates PRGF survival and prevention of H_2_O_2_ cytotoxicity. (a) Human ASCs were treated with 100 *μ*M H_2_O_2_ for 24 h in the presence or absence (0%) of 2.5% PRGF or 2.5% HS, and the cell viability was analyzed by MTT assay. The significant reduction on cell viability induced by hydrogen peroxide was prevented by 2.5% of PRGF. ^∗^*P* < 0.05 versus 0 *μ*M H_2_O_2_ and ^#^*P* < 0.05 versus 100 *μ*M H_2_O_2_. (b) Preincubation with 10 *μ*M of the AKT inhibitor (Calbiochem, VIII 124018) abolished the cell protective effect of PRGF of the cytotoxic effects of 100 *μ*M H_2_O_2_. ^∗^*P* < 0.05 versus 0% PRGF. (c) FACS analysis of annexin V showed the percentage of apoptotic cells according to each treatment. ^#^*P* < 0.05 versus 2.5% PRGF. (d) Western blot analysis of total protein lysates of the human ASCs treated with 2.5% PRGF (+) or 0% PRGF (−) in the presence of 100 *μ*M, preincubated or not for 30 min with 10 *μ*M AKT inhibitor for 24 h. Activation or cleavage of the apoptotic protein PARP is confirmed when two bands visible. *β*-Actin was employed as a loading control. Representative blots of three different experiments are shown; ^∗^*P* < 0.05.

Acute bouts of oxidative stress stimulate cell proliferation in ASCs [41]; however, high doses, or extended exposure to low doses of reactive oxygen species, dramatically reduce ASC viability [42]. Treatment with 100 *μ*M H_2_O_2_ for 24 h was significantly toxic to human ASCs in the absence of growth factors (Figure 4(a)). Consistent with the perceived cytoprotective effect of PRGF, quantification of cell viability revealed that in the presence of 2.5% PRGF, but not 2.5% HS, PRGF significantly prevented the cell death that was significantly compromised by the addition of 100 *μ*M H_2_O_2_ (Figure 4(a)).

Pharmacological inhibition of AKT by the preincubation for 30 minutes with 10 *μ*M of the Inhibitor VIII 124018 (Calbiochem) blocked the protective effect of 2.5% of PRGF on ASCs exposed to a cytotoxic H_2_O_2_ dose (Figure 4(b)). Furthermore, the percentage of the population of apoptotic cells, quantified by the expression of annexin V, was determined in the presence or absence of PRGF and when treated with H_2_O_2_ alone or with AKT inhibitor. The results demonstrated that PRGF induced cell survival via activation of AKT since the inhibition of AKT leads to a significant population of annexin V-positive cells, including those in the presence of PRGF (Figure 4(c)).

ASCs require of trophic factors since in the absence of PRGF or HS, there was an increase of basal activation of apoptosis as evidenced by the cleavage of PARP, a protein associated with the induction of apoptosis signaling [43] (Figure 4(d)). Moreover, the presence of PRGF blocked the induced PARP cleavage when ASCs were treated with 100 *μ*M H_2_O_2_. In the presence of PRGF, phosphorylated AKT (active form) and cyclin D (downstream effector of AKT) are expressed when PARP has not been cleaved.

## 4. Discussion

ASCs and PRGF have been shown as promising therapeutic alternatives for tissue repair in mesoderm-related tissues, like, for instance, in the repair of the damaged cartilage in osteoarthritis. ASCs are easily expanded in culture and are capable of differentiating by their multipotent nature in various mature cell lineages allowing to reproduce in vitro processes such as osteogenesis, chondrogenesis, or adipogenesis [44]. However, still there is much uncertain information about the ex vivo amplification, behaviour of the transplanted cells in terms of cell survival, tissue integration, or cell differentiation in a hostile microenvironment. Nowadays, PRGF offers an autologous source of known regenerative properties being able to synergistically improve the benefits of ASC treatments. PRGF favors ASC proliferation [7, 8] and is able to stimulate undifferentiated stem cell differentiation for tissue regeneration [17, 45]. Undifferentiated stem cells migrate to the concentration of PRGF growth factor gradients, and the growth factors trigger proliferation of these cells once they are at the site of administration [46]. The combinatory use of PRGF and ASCs has already shown improved benefits in a number of tissue repair process, including osteoarthritis or by accelerating ossification of fractures [47–49].

Cell transplantation efficiency is directly depending on cell survival, cell aging, and cell fate specification. Rejuvenation of aged progenitor cells has been largely reported by exposure to a young or regenerative environment by reducing the cell senescence [50]. Recent studies suggest that blood from young donors could reverse age-related diseases based on experiments of blood transference from young mice which could rejuvenate aged tissues in older animals [50–52]. After analyzing several circulating factors, Loffredo et al. [52] suggested that GDF11 could be responsible for these effects. Recently, Bueno et al. [53] have shown that GDF11 is at least ten times more concentrated in platelet lysate than in serum or plasma, indicating that GDF11 is stored in platelets contributing most probably to the differential effect of PGRF on cell proliferation, cell differentiation, or cell aging [54]. We have observed a significant dependency on the AKT signaling activation with survival responses in ASCs in the absence of growth factors and by oxidative damage. PRGF by inducing phosphorylation of AKT significantly improves cell survival of ASCs when are exposed to proapoptotic concentrations of hydrogen peroxide. The hydrogen peroxide is employed to reproduce the oxidative stress found at the injury and transplantation area. The inflammatory-related oxidative stress in fact compromises the cell function and survival of the entire joint, including the cartilage in the osteoarthritic patients [55]. In this context, PRGF treatment would prevent cell death associated to oxidative stress by inducing prosurvival signals in the joint by activation of AKT [56].

The efficiency of ASCs for osteoarthritis has been already probed with significant functional improvements. Dogs provide a suitable translational model for further clinical research on osteoarthritis, with close histopathological and functional features [57]. We recently showed in a randomized study performed in canine patients, with moderate to severe osteoarthritis, that intra-articular transplantation of ASC provided a significant joint functional improvement, reducing dog's pain and improving physical function up to six months [58, 59]. In fact, ASCs from canine samples were also tested in vitro, in response to PRGF, and we found a similar response to cell proliferation, survival, and differentiation found in human ASCs (Supplementary Figure). Based on in vitro experimentation, significant improvements would be expected for activated ASCS by PRGF stimulation; however, further in vivo analysis is needed to disclose it. Thus, ASCs in culture pretreated with PRGF before transplantation may confer improved survival, proliferation, and antiapoptotic capabilities and render the cells a powerful source for cell therapy and tissue regeneration. Canine and human ASCs showed a comparable response to PRGF of cell proliferation, cell differentiation, and AKT induction. Thereby, dogs provide a suitable translational model for further clinical research on ASC-based treatments.

## 5. Conclusion

PRGF treatment is a potent stimulator of ASCs and can be used as an autologous preconditioning agent to enhance the therapeutic potential of ASC transplantation.

## Supplementary Material

Supplementary Figure 1 Canine ASC were cell cultured with FBS or canine PRGF at growing concentration (1, 2.5, 5 or 10%) or in the absent of growth factors (0) for 24 hours and subjected to cell viability test analysis. 10% PRGF induced a significant difference on the cell numbers in comparison with 10% of FBS; ∗p>0.05 vs 0%, $p>0.05 vs 10% FBS. Supplementary Figure 2 Canine ASC were induced to differentiate toward the three mesodermal lineages in the presence or absence of canine 2.5% PRGF. A) Adipogenesis was evidenced by intracellular lipid content by Oil Red O staining; B) Osteogenesis was detected by calcium deposits visualized by Alizarin Red staining and phalloidin (green) and Cx43 expression (red); C) Chondrogenesis was analysed by Alcian blue staining (marker of the proteoglycan aggrecan deposits), and immunostaining of Sox9 (green; marker of chondrogenesis and chondrocyte differentiation).
