# Supplementary figures and images for: Plasma Rich in Growth Factors Induces Cell Proliferation, Migration, Differentiation, and Cell Survival of Adipose-Derived Stem Cells
Source: Stem Cells Int. 2017 Nov 15;2017:5946527. doi: 10.1155/2017/5946527 (PMC5705873; doi:10.1155/2017/5946527)

**
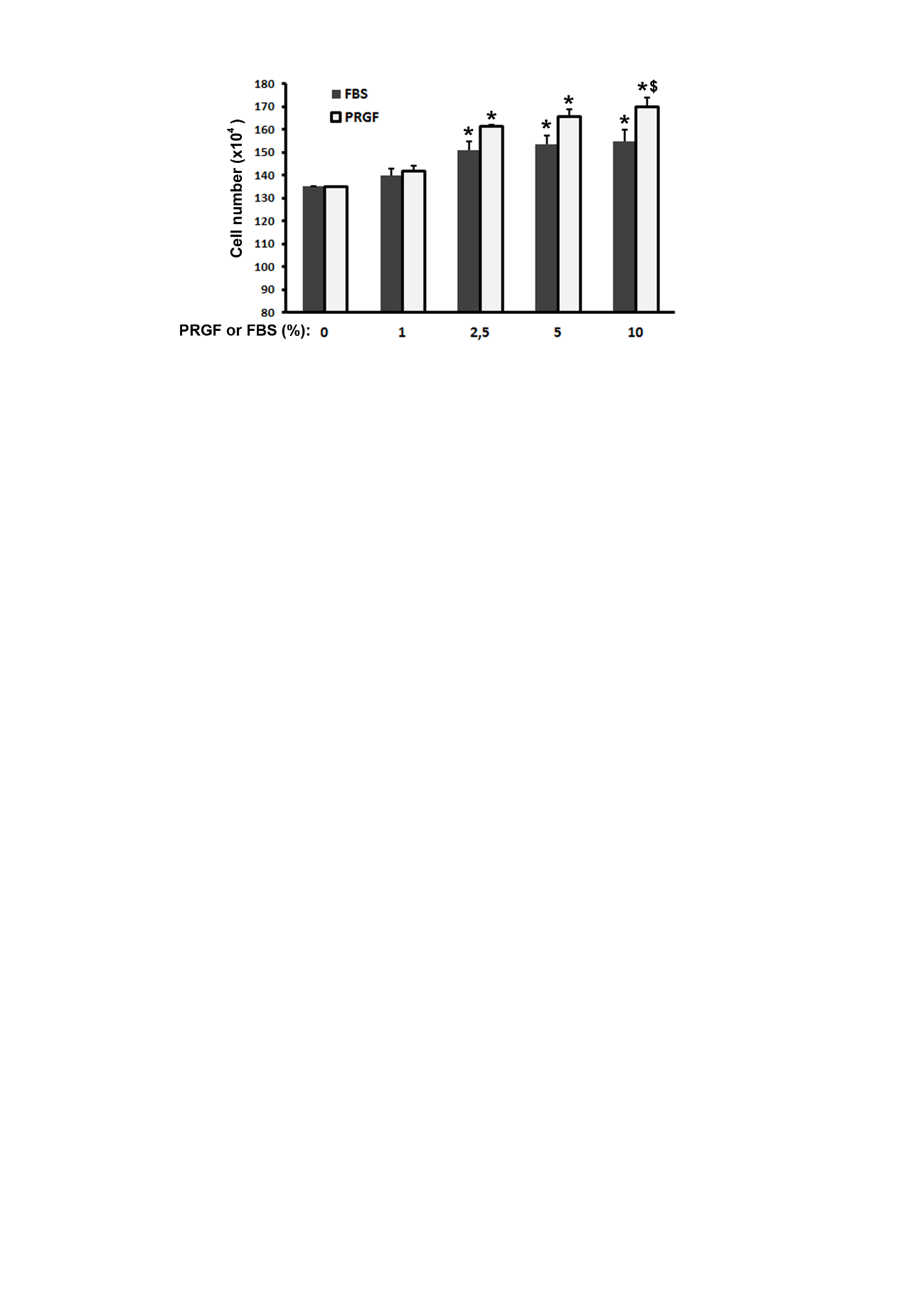
**

**Supplementary Figure 1**

**
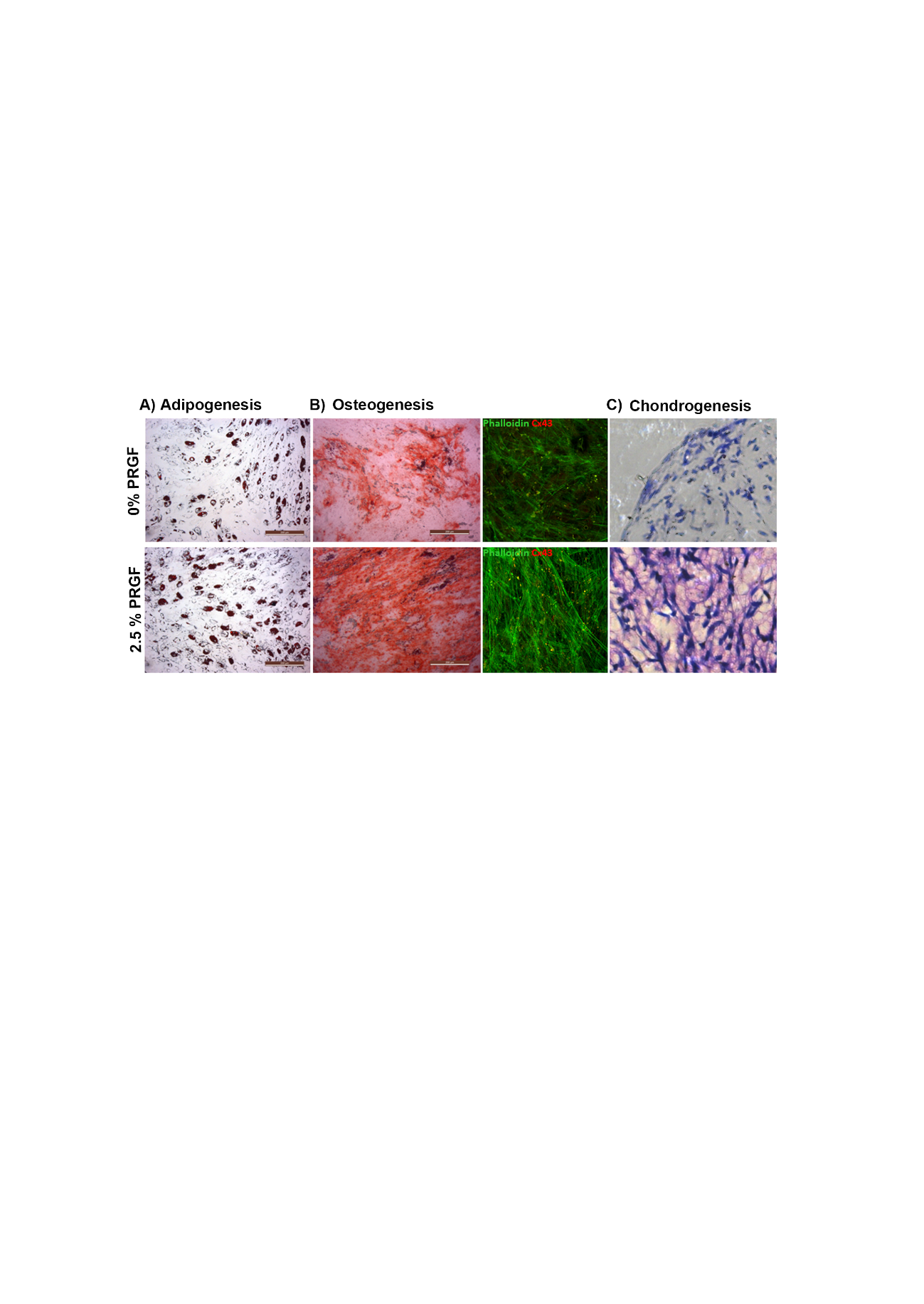
**

**Supplementary Figure 2**

Supplement: Supplementary file 1 — Supplementary Figure 1 Canine ASC were cell cultured with FBS or canine PRGF at growing concentration (1, 2.5, 5 or 10%) or in the absent of growth factors (0) for 24 hours and subjected to cell viability test analysis. 10% PRGF induced a significant difference on the cell numbers in comparison with 10% of FBS; ∗p>0.05 vs 0%, $p>0.05 vs 10% FBS. Supplementary Figure 2 Canine ASC were induced to differentiate toward the three mesodermal lineages in the presence or absence of canine 2.5% PRGF. A) Adipogenesis was evidenced by intracellular lipid content by Oil Red O staining; B) Osteogenesis was detected by calcium deposits visualized by Alizarin Red staining and phalloidin (green) and Cx43 expression (red); C) Chondrogenesis was analysed by Alcian blue staining (marker of the proteoglycan aggrecan deposits), and immunostaining of Sox9 (green; marker of chondrogenesis and chondrocyte differentiation). [file 5946527.f1.docx]
